# The Effect of Modulation Ratio of Cu/Ni Multilayer Films on the Fretting Damage Behaviour of Ti-811 Titanium Alloy

**DOI:** 10.3390/ma10060585

**Published:** 2017-05-26

**Authors:** Xiaohua Zhang, Daoxin Liu, Xiaoying Li, Hanshan Dong, Yuntao Xi

**Affiliations:** 1 Institute of Corrosion and Protection, School of Aeronautics, Northwestern Polytechnical University, Xi’an 710072, China; liudaox@nwpu.edu.cn; 2 School of Metallurgy and Materials, The University of Birmingham, Birmingham B15 2TT, UK; X.LI.1@bham.ac.uk (X.L.); H.DONG.20@bham.ac.uk (H.D.); 3 Oil & Gas Technology Research Institute, Changqing Oilfield Company, Xi’an 710018, China; xytao_cq@petrochina.com.cn

**Keywords:** fretting fatigue, fretting wear, multilayer film, titanium alloy, modulation ratio

## Abstract

To improve the fretting damage (fretting wear and fretting fatigue) resistance of Ti-811 titanium alloy, three Cu/Ni multilayer films with the same modulation period thickness (200 nm) and different modulation ratios (3:1, 1:1, 1:3) were deposited on the surface of the alloy via ion-assisted magnetron sputtering deposition (IAD). The bonding strength, micro-hardness, and toughness of the films were evaluated, and the effect of the modulation ratio on the room-temperature fretting wear (FW) and fretting fatigue (FF) resistance of the alloy was determined. The results indicated that the IAD technique can be successfully used to prepare Cu/Ni multilayer films, with high bonding strength, low-friction, and good toughness, which yield improved room-temperature FF and FW resistance of the alloy. For the same modulation period (200 nm), the micro-hardness, friction, and FW resistance of the coated alloy increased, decreased, and improved, respectively, with increasing modulation ratio of the Ni-to-Cu layer thickness. However, the FF resistance of the coated alloy increased non-monotonically with the increasing modulation ratio. Among the three Cu/Ni multilayer films, those with a modulation ratio of 1:1 can confer the highest FF resistance to the Ti-811 alloy, owing mainly to their unique combination of good toughness, high strength, and low-friction.

## 1. Introduction

Characterised by high specific strength and excellent mechanical properties, titanium (Ti) alloys are important materials for space and aeronautic applications [1,2]. However, these alloys suffer from fretting fatigue (FF) damage, owing to their low thermal conductivity and high coefficient of friction. This can affect the safety and reliability of Ti components [3,4]. Fretting fatigue is a type of fatigue damage that occurs between two surfaces having oscillatory relative motion of small amplitude under the combined actions of cyclic bulk stresses, cyclic contact stresses, and reciprocating sliding [5]. Therefore, FF damage depends on the fretting wear (FW) and fatigue resistance of the material, and surface modification or coating is considered a promising method for combating this damage in Ti alloys. 

Many attempts, including methods such as shot peening, laser shock processing, low plasticity burnishing, plasma alloying, ion irradiation, physical vapour deposition (PVD), thermal spraying, and ion beam enhanced deposition, have been made to improve the FF properties of Ti alloys [6,7,8,9]. However, improvement of the fretting fatigue resistance (FFR) via surface treatments is difficult, because rather than combined improvement, these treatments yield either improved anti-friction or anti-fatigue properties. For example, surface-hardening treatments can effectively improve the wear resistance of materials, but (in many cases) this increased surface hardness is realized at the expense of the fracture toughness and, hence, the fatigue properties [10]. Our research has shown that plasma nitriding is detrimental to the FFR of Ti-6Al-4V alloy, although this surface treatment results in improved hardness and wear resistance of the material [11]. Achieving a suitable combination of hardness, wear resistance, fatigue resistance, toughness, and low-friction is essential for many applications, and this combination may be realized via multilayered films. Therefore, understanding the deposition effect of multilayer films on the FFR of Ti alloys would be helpful in this regard.

Compared with monolayer films, multilayer films have higher hardness, wear resistance, and fracture toughness [12,13], owing to the inhibition or retardation of dislocation movement and atom diffusion by the numerous interfaces in these films [14,15]. In particular, supermodulus and superhardness properties of the multilayer films can be achieved when the modulation period is limited to the nanometer scale [16,17]. The properties of these films depend on several factors, such as the chemical constitution, modulation period, and modulation ratio of the films. The high-temperature stability and the toughness of the films can be enhanced by designing a suitable chemical constitution. Moreover, the anti-friction properties, hardness, and the ability to inhibit micro-crack propagation can be improved by optimising the modulation period of the films.

Previous research has indicated that the fretting damage resistance of Ti alloys can be significantly improved by using Cu/Ni multilayer films with different (i.e., repeated) modulation periods [18]. However, for a fixed modulation period, the modulation ratio (i.e., the ratio of Ni and Cu thickness within one modulation period) can also be varied. The modulation ratio of multilayer films also affects the corresponding hardness and sliding wear properties [19]. However, the effect of the modulation ratio on the FF and FW resistance of Ti alloys remains unexplored. Knowledge of this effect will advance scientific understanding and provide technical information for the optimal design of Cu/Ni multilayer films. Therefore, in the present study, the effect of IAD Cu/Ni multilayer films on the FF and FW resistance of Ti alloys was investigated and the mechanisms involved were discussed. Films with a fixed modulation-period thickness of 200 nm and different modulation ratios (Cu:Ni = 3:1, 1:1, and 1:3) were considered.

## 2. Materials and Methods 

### 2.1. Materials

**Figure 1:**
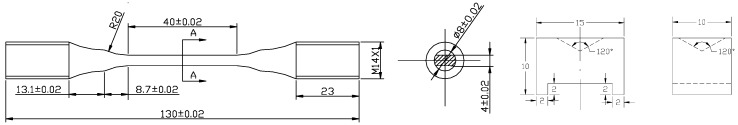
Schematic of FF specimens and fretting pads. (Unit: mm).

A Ti-811 alloy with a composition of 7.9% Al, 1.0% Mo, 0.99% V, 0.05% Fe, 0.1% C, 0.01% N, 0.001% H, 0.06% O, and the balance, Ti, was used in this study. The material was first annealed at 910 °C for 1 h, air cooled, annealed at 580 °C for 8 h, and air cooled again. This treatment yielded a microstructure consisting of an equiaxed α-phase and an intergranular β-phase. The alloy had the following mechanical properties: ultimate strength = 931 MPa, yield strength = 890 MPa, elongation = 23% and area reduction = 46%, fatigue strength = 540 MPa, and fracture toughness = 78 MPa·m^1/2^. FF specimens and fretting pads were cut from Ø 16 mm Ti-811 alloy bars (see Figure 1 for the dimensions of the test specimens and contact pads).

### 2.2. Research Methods

Cu/Ni multilayer films with different modulation ratios were deposited via ion-assisted magnetron sputtering in a PIEMAD-03 multifunction apparatus (Beiyu Vacuum Equipment Co. Ltd., Shenyang, China) consisting of two magnetron sputtering targets, four multi-arc cathodes, and an assisted ion source. Prior to film deposition, the specimen was finely ground using 1200 grit SiC paper, ultrasonically cleaned with acetone, and then cleaned for 20 min with a 3 keV Ar ion beam (flux: 300 μA/cm^2^). Afterwards, a ~1 μm Cu interlayer was deposited to enhance the adhesion of the multilayer films to the substrate. The Cu and Ni sublayers were separately produced, via magnetron sputtering, from 99.99% Cu to 99.99% Ni targets, respectively. The thickness and the modulation period of all the films were 10 μm and 200 nm, respectively. Furthermore, the modulation ratio of Ni to Cu of the films was controlled to 1:3, 1:1, and 3:1 by controlling the sputtering time of the two targets. 

The surface morphology of the Cu/Ni multilayer films was observed using a field emission scanning electron microscope (FESEM; JOEL 7000, Tokyo, Japan). The corresponding fretted zone was characterized via energy dispersive X-ray (EDX) analysis (JOEL Ltd., Tokyo, Japan) using a spectrometer attached to the FESEM. The micro-hardness was measured (load: 0.245 N, loading time: 20 s) using an HV-1000 micro-hardness tester (Metallurgical Equipment Co. Ltd., Shanghai, China) equipped with a Knoop indenter. A scratch instrument was used to measure the bonding strength between the multilayer films and the substrate. The critical bonding strength (Lc) was determined when the films began to spall from the substrate. An in-house-fabricated repetitive indent tester, equipped with a rectangular pyramid indenter was operated for 1 × 10^4^ cycles at a repeating applied load of 60 N. The impression morphology was observed by a WD300LCS optical microscope (Cewei Photoelectric Technology Co. Ltd, Xi’an, China). The compressive residual stress was measured with a LXRD-MG2000 X-ray diffraction (PROTO Manufacturing Ltd., Oldcastle, ON, Canada) residual stress analyser. These measurements were performed with a Cu radiation source (λ = 1.5406 Å) operating at 40 kV and 40 mA, and the strain associated with the Cu (331) peak at 2θ = 136° and the Ni (311) peak at 2θ = 144° was measured.

A ball-on-flat geometric configuration was used to evaluate the fretting wear (FW) performance of the Cu/Ni multilayer films. All FW tests were carried out on a SDS100 electro-hydraulic servo machine, which is fully computer-controlled [11]. The flat with films was fixed and the Ti-811 ball reciprocated. The diameter of the ball is 10 mm. Testing was performed under the following conditions: frequency 120 Hz, slip amplitude 36 μm, number of cycles 1 × 10^4^, and normal force 200 N (maximum Hertzian normal pressure is 355 MPa).

**Figure 2:**
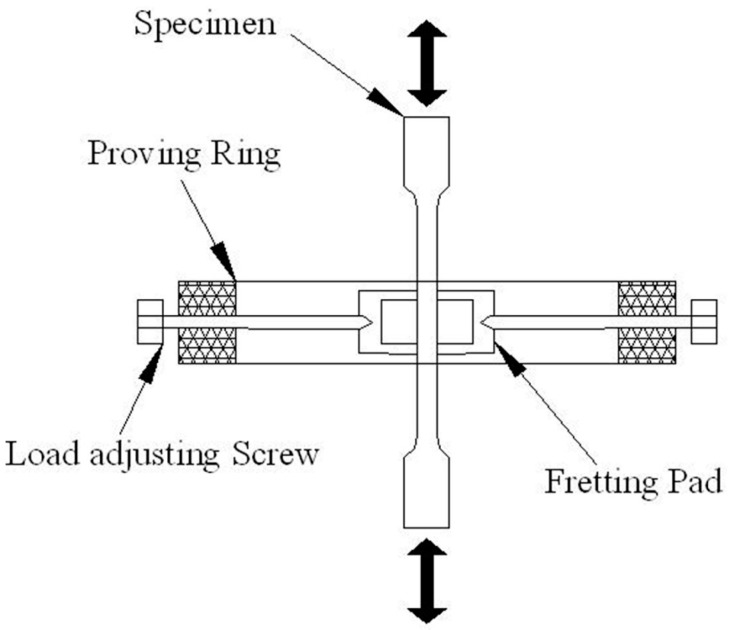
Schematic illustration of the fretting fatigue test setup.

In addition, FF tests were performed at room temperature on a PLG-100C high-frequency fatigue machine (see schematic in Figure 2 [18]). An average contact stress of 85 MPa was generated in the 2 mm × 6 mm contact area formed by the rectangular pad foot and the fatigue specimen. The axial loading stress was 700 MPa, in a sinusoidal form at 110 Hz, with a stress ratio of 0.1. The FF life was taken as the average of the values obtained for three specimens. 

## 3. Results

### 3.1. Hardness and Toughness of the Cu/Ni Multilayer Films

**Figure 3:**
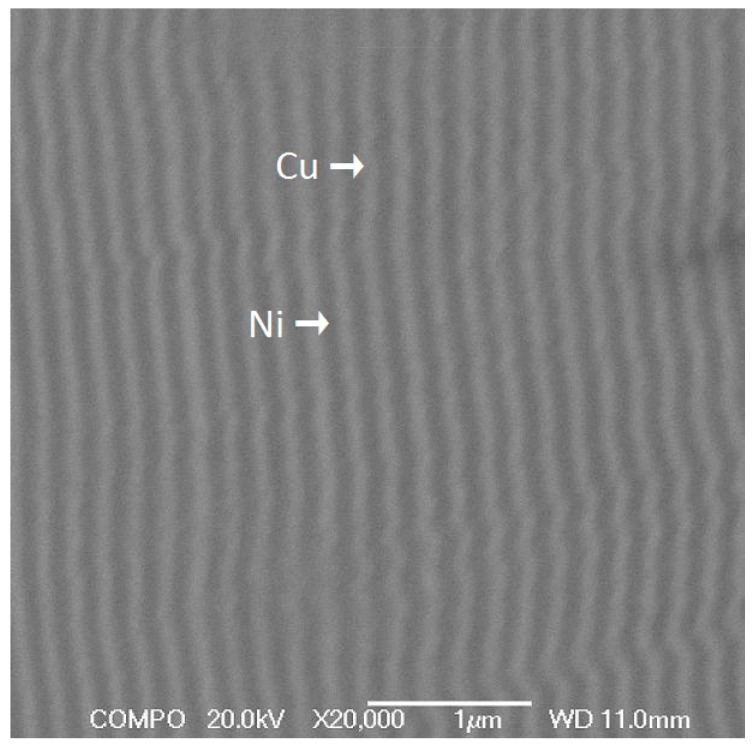
Backscattering electron microscopy (BEM) image of a Cu_100nm_Ni_100nm_ film.

The micrograph in Figure 3 shows a typical cross-section of the deposited Cu/Ni multilayer films. The alternating bright and dark lines of this interspace-free film correspond to Cu layers and Ni layers, respectively.

**Figure 4:**
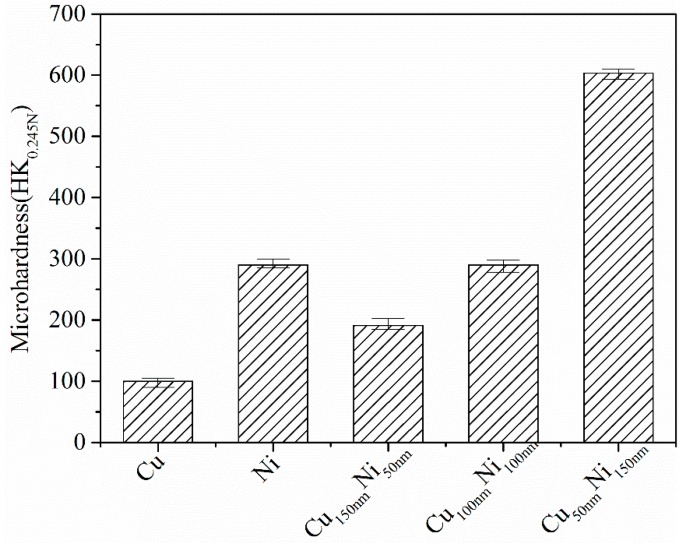
Microhardness of the Cu film, Ni film, and Cu/Ni multilayer films with different modulation ratios.

The microhardness results obtained for the various surface films are summarised in Figure 4. As the figure shows, the Ni monolayer is approximately three times harder than the Cu monolayer, and the Cu/Ni multilayer films are all harder than the pure Cu film. For the same modulation period, the microhardness of the multilayer films increased with the increasing layer-thickness ratio of Ni to Cu. The multilayer films with a modulation ratio of 1:3 (Cu_50nm_Ni_150nm_) had the highest hardness of all the investigated films (including the pure Ni film). 

During scratch tests under a 100-N scratch load, the multilayer films were all resistant to spalling, indicative of strong bonding between the films and the substrate. 

**Figure 5:**
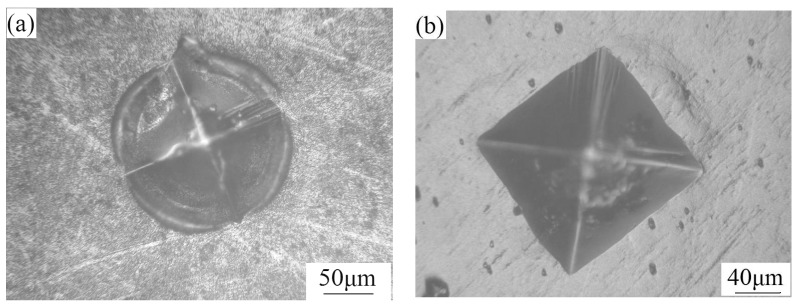

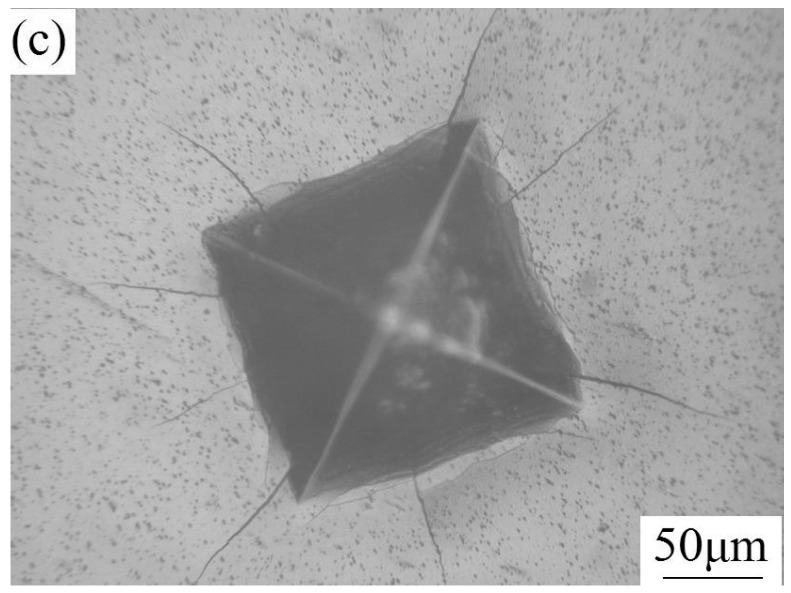
Morphology of the indents resulting from repeated indentation of the Cu/Ni multilayer films with modulation ratio of (**a**) 3:1 (Cu_150nm_Ni_50nm_); (**b**) 1:1 (Cu_100nm_Ni_100nm_); and (**c**) 1:3 (Cu_50nm_Ni_150nm_).

The surface morphology after repeated indentation of three Cu/Ni multilayer films is shown in Figure 5. The region around the indentation underwent severe plastic deformation, owing to the low hardness (see Figure 5a) and, hence, low load-bearing capacity of the multilayer films with a modulation ratio of 3:1 (Cu_150nm_Ni_50nm_). However, the absence of spallation or cracking is indicative of the high film–substrate bonding strength and toughness. The multilayer films with modulation ratios of 1:1 (Cu_100nm_Ni_100nm_) and 1:3 (Cu_50nm_Ni_150nm_) had relatively high hardness and load-bearing capacity and, therefore, the indenter perimeter of these films underwent only modest deformation. In addition, the regions within, and around, the indent in Cu_100nm_Ni_100nm_ film were crack-free, consistent with the relatively high toughness of the film. However, many radial cracks appeared at the sharp corners and along the edges of the indent in the Cu_50nm_Ni_150nm_ film, indicating that this film, although resistant to spallation, has very low toughness.

The results of the residual stress measurements on the films are summarised in Table 1. As the table shows, these stresses are all compressive and the stress in the Cu/Ni multilayer films are higher than that in the pure Cu or Ni film. 

### 3.2. Fretting Wear

**Figure 6:**
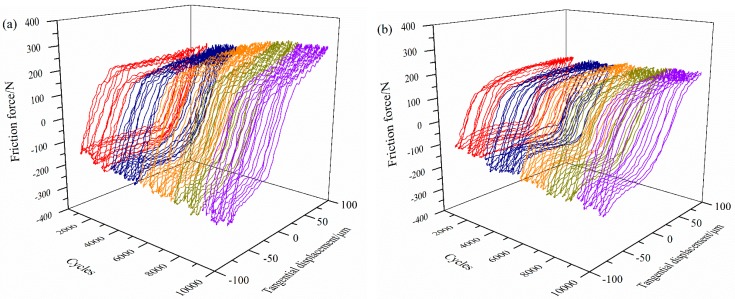

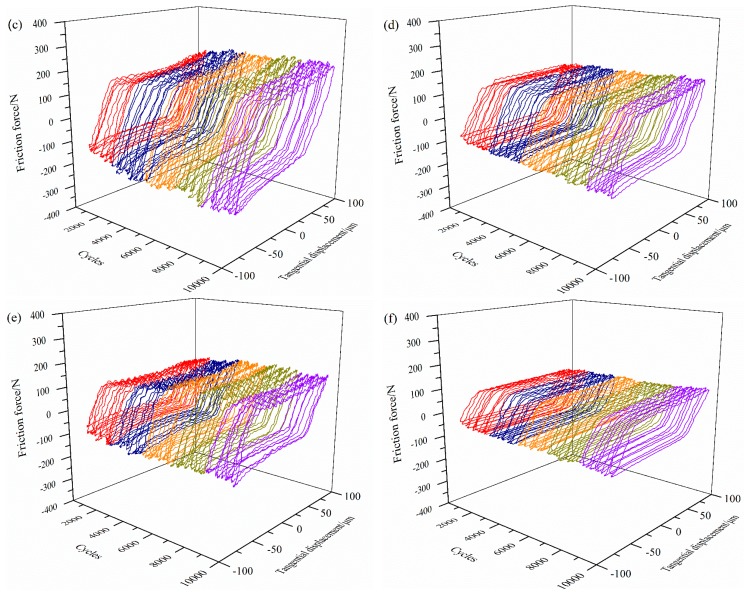
Fretting (Q-D-N) logs of the Cu/Ni multilayer films and Ti-811 substrate subjected to fretting wear (FW) tests: (**a**) Ti-811; (**b**) Ni; (**c**) Cu; (**d**) Cu_150nm_Ni_50nm_; (**e**) Cu_100nm_Ni_100nm_; and (**f**) Cu_50nm_Ni_150nm_.

**Figure 7:**
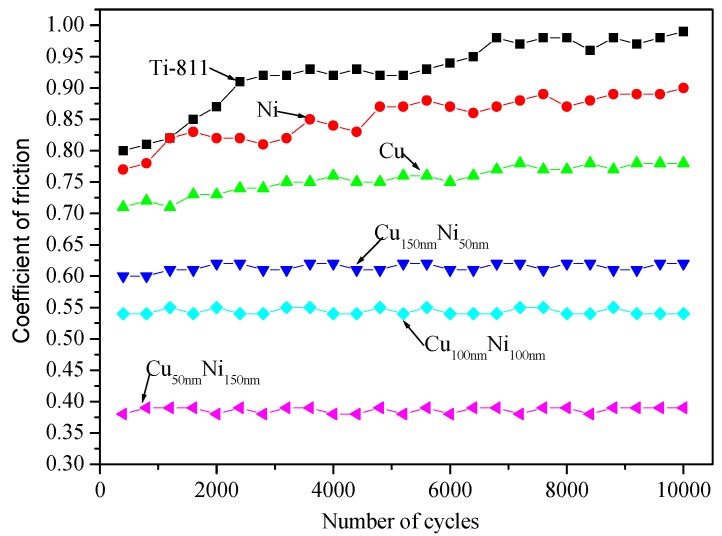
Variation of coefficient of friction during FW tests.

The fretting logs of the Cu/Ni multilayer films developed in this study, pure Cu and pure Ni films, and the Ti-811 substrate, are shown in Figure 6. The Q-D-N (Q: friction force, D: relative displacement, and N: number of cycles) curve of the substrate was shaped initially like a regular parallelogram, but became slender in subsequent stages (see Figure 6a). This indicated that the friction force increased and the fretting contact region of the substrate was in the partial slip condition. The Q-D-N curve of the Cu/Ni multilayer films formed a parallelogram (Figure 6d–f), consistent with the gross-slip state of the fretting contact region. This is attributed to the low coefficient of friction of the Cu/Ni multilayer films (Figure 7). 

**Figure 8:**
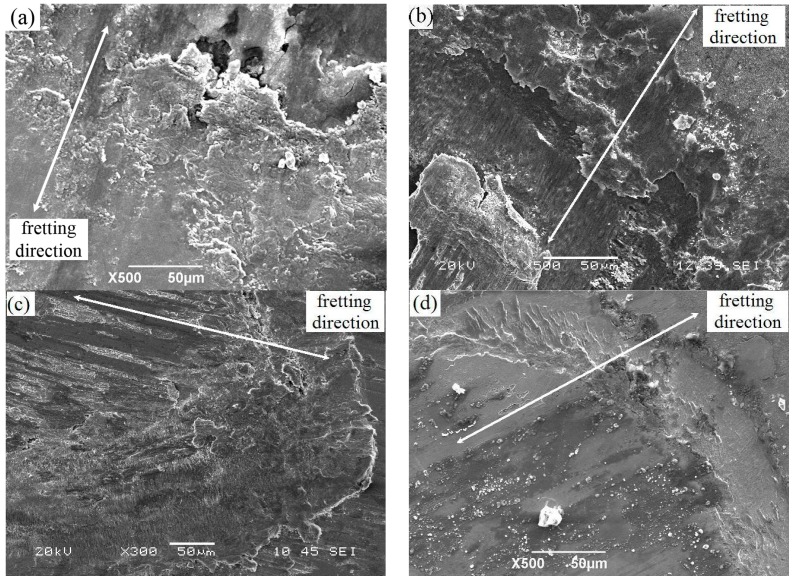
Post-fretting-wear SEM images of (**a**) Ti-811 and Cu/Ni multilayer films of (**b**) Cu_150nm_Ni_50nm_; (**c**) Cu_100nm_Ni_100nm_; and (**d**) Cu_50nm_Ni_150nm_.

Scanning electron micrographs (Figure 8) of the FW morphology reveal delamination and cracking on the surface of the Ti-811 alloy (Figure 8a). Material removed from the surface via delamination produced wear debris during the fretting process, consistent with strong adhesion between the contact surface of the alloy specimen and the alloy ball. This is attributed to the partial slip state of the alloy, where the highest friction force occurs during the FW process. Multilayer films with a modulation ratio of 3:1 (Cu_150nm_Ni_50nm_) exhibited more severe delamination (Figure 8b) than their 1:1 (Cu_100nm_Ni_100nm_) counterparts (Figure 8c), which had higher hardness, load-bearing capacity, and anti-friction capacity. Possibly owing to the high hardness and low friction of the 1:3 (Cu_50nm_Ni_150nm_) films, little debris was generated with a further reduction of the FW associated with this film (Figure 8d). In summary, for the same modulation period, the friction and FW of the Cu/Ni multilayer films decreased with the increasing layer-thickness ratios of Ni to Cu. 

### 3.3. Effect of Cu/Ni Multilayer Films on the FF Life of Ti-811 Alloy

A comparison of the fretting fatigue life (FFL) of the Ti-811 alloy coated with different surface films reveals that the films all result in improved FFL of the alloy (Table 2). The FFL improved by factors of 1.94, 0.22, 1.07, 3.94, and 2.34 owing to the pure Cu, pure Ni, and 3:1 (Cu_150nm_Ni_50nm_), 1:1 (Cu_100nm_Ni_100nm_), and 1:3 (Cu_50nm_Ni_150nm_) Cu/Ni multilayer films, respectively. The FFR did not increase monotonically with the Cu ratio of the multilayer films. As the results show, the best FFR is obtained for multilayer films with a modulation ratio of 1:1 (Cu_100nm_Ni_100nm_).

**Figure 9:**
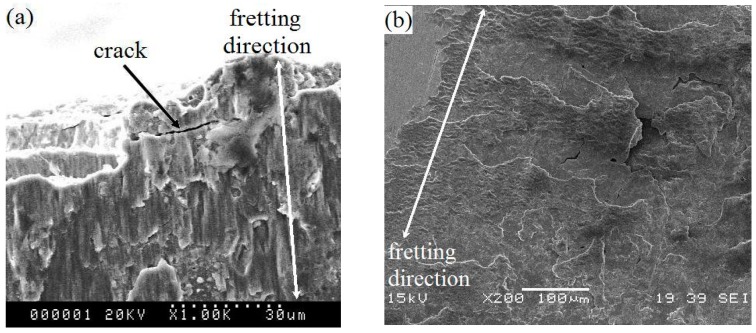

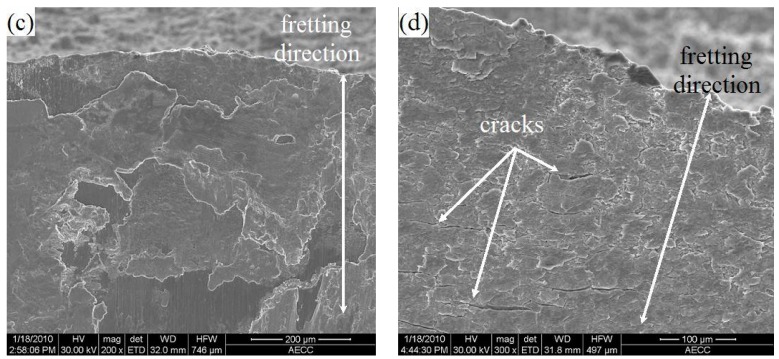
SEM images of fretting-fatigue-tested surfaces of the Ti-811 alloy (**a**) only and with Cu/Ni multilayer films of (**b**) Cu_100nm_Ni_100nm_; (**c**) Cu_150nm_Ni_50nm_; and (**d**) Cu_50nm_Ni_150nm_.

**Figure 10:**
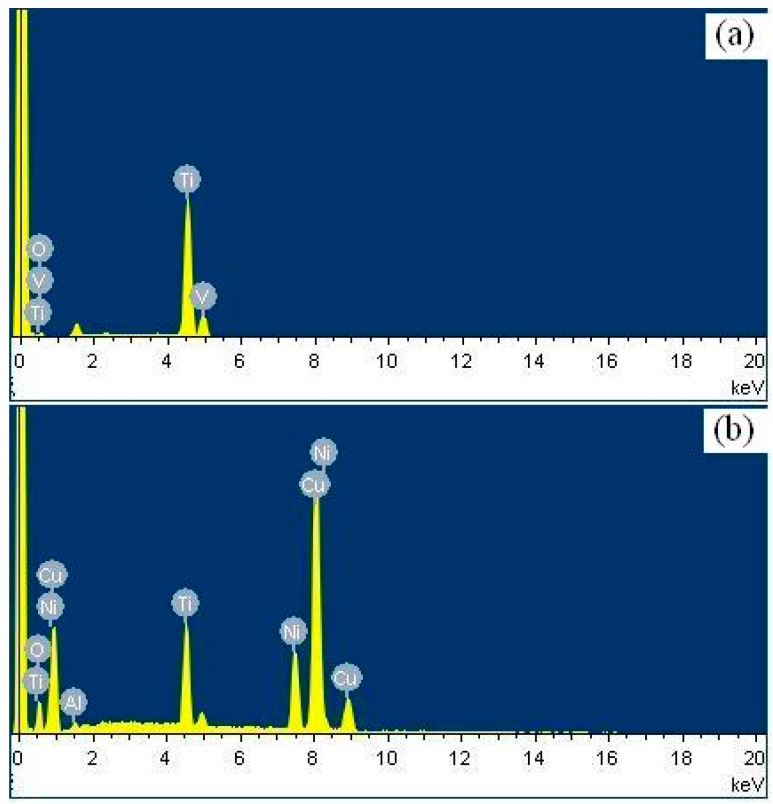
EDX pattern at the fretted zone of (**a**) Ti-811 and (**b**) Cu_100nm_Ni_100nm_.

The FF damage morphologies of the Ti-811 alloy coated with different films are shown in Figure 9. The FF damaged surface or FF scar of the alloy consists of cracks and severe adhesion and deformation features (Figure 9a). This possibly results from the strong adhesion between the fretting pad and the specimen, which are both composed of the same Ti material. Furthermore, at small slip amplitude, cracks were easily generated between the slip and surrounding non-slip regions, owing to stress concentration. The tangential friction force, which was quite large, varied significantly during slip and acted iteratively on the contact area, i.e., the boundary between the slip and non-slip area. A very large partially compressive stress and the maximum tensile stress occurred in front of, and behind, the slip area, respectively. Severe plastic deformation and partial wear occurred at the surface of the material. Moreover, accelerated crack initiation under FF conditions resulted in micro-crack formation in the contact area. The multilayer films with a modulation ratio of 1:1 (Cu_100nm_Ni_100nm_) remained intact during the FF process, owing to their relatively high hardness, low friction, and high toughness. Examination of the fretting region revealed fatigue delamination of the constituent monolayers (Figure 9b). Owing to their low hardness, multilayer films with a modulation ratio of 3:1 (Cu_150nm_Ni_50nm_) were easily removed during fretting and, in this case, the Ti-811 alloy substrate was severely damaged (Figure 9c). Cracks formed within the FF scar (Figure 9d) of the 1:3 (Cu_50nm_Ni_150nm_) films, although these films had the highest hardness, lowest friction, and best FW resistance among the three multilayer films. Figure 10 and Table 3 show the results of EDX analyses of the fretted zones shown in Figure 9. Oxygen was present in each zone, consistent with a possible increase in temperature and the occurrence of an oxidation process during FF. The oxygen content of the fretted zone of the Ti-811 alloy was higher than that of the Cu/Ni multilayer films, indicating that intense oxidation occurred in this zone, due to strong adhesive wear. This further indicated that the good solid-lubrication capacity of the multilayer films contributed to reductions in both the wear and the degree of oxidation. Furthermore, Ti in the fretted zone of the multilayer films was transferred from the fretting pad. The EDX results indicated that in addition to mechanical interactions, physical and chemical interactions may also occur at the interface between the fretting pad and the specimen.

## 4. Discussion

### 4.1. Hardness of Cu/Ni Multilayer Films 

As shown in Figure 4, for the same modulation period of 200 nm, the microhardness of the Cu/Ni multilayer films increased with increasing Ni to Cu ratios. This is attributed to the fact that Ni is considerably harder than Cu. The hardness of the multilayer films with a modulation ratio of 3:1 (Cu_150nm_Ni_50nm_) can be predicted by the theory of composite hardness [20]. However, the hardness of the multilayer films with a modulation ratio of 1:1 (Cu_100nm_Ni_100nm_) and 50% Cu is almost the same as that of pure Ni (Figure 4). Moreover, the hardness of the films with a modulation ratio of 1:3 (Cu_50nm_Ni_150nm_) is double that of Ni and six times that of Cu. This result is inconsistent with the classical composite hardness theory. 

The superhardness of the Cu/Ni multilayer films may be attributed to: (1) nanoscopic features, the interfaces in the multilayer films can act as pinning sites for dislocation movement [21]; and (2) microscopic features, the film probably consisted of nanometer-sized grains. 

### 4.2. Residual Stress of Cu/Ni Multilayer Films

A compressive residual stress, resulting from atomic peening, was generated in the films by ion bombardment during deposition. The energetic ions resulted in the incorporation of atoms into spaces in the growing film, with volumes smaller than the typical atomic volume, leading to expansion of the film outwards from the substrate. While, in the plane of the film, the film is unable to expand. 

Owing to the alternating stress field in the Cu/Ni multilayer films, the compressive residual stress in these films is higher than that in the pure Cu or Ni film. The crystallographic lattice constant of Cu is greater than that of Ni (3.608 vs. 3.517 Å). Therefore, the Cu layers and Ni layers experienced a compressive stress and a tensile stress, respectively, in the Cu/Ni interfaces, and an alternating stress field with a nanometer-scale modulation period was thereby generated. The residual compressive stress in the multilayer films increased, owing to multiple alternating stress fields generated in the interfaces. For the same modulation period, the compressive residual stress in the Cu layer increased gradually with increasing layer-thickness ratios of Ni to Cu, whereas the corresponding stress in the Ni layer decreased gradually. This possibly resulted from the difference in layer thickness, since residual stress decreases with an increasing layer thickness [22]. 

The compressive residual stress is associated with crack closure, particularly with the retardation of crack propagation in the initial stages. This stress also reduces the effective tensile stresses induced by cyclic loading and fretting contact, thereby delaying, or even preventing, crack initiation.

### 4.3. Friction of Cu/Ni Multilayer Films 

As shown in Figure 6, the dynamic friction force of the Ti-811 substrate can be reduced by using pure Ni and pure Cu monolayers. This reduction can be explained via the adhesive theory [23] based on metallurgical compatibility, which is determined by the solid solubility between the articulating surfaces. In this study, a Ti ball was used for the FW tests and the solid solubility of this ball in Ti, Ni-coated Ti, and Cu-coated Ti specimens is 100, >1, and <1%, respectively. Therefore, the adhesion between the Ti ball and the specimen may be arranged in descending order as follows: Ti ball-Ti flat, Ti ball-Ni coated flat, and Ti ball-Cu coated flat. 

The dynamic friction force of each multilayer film is lower than that of the uncoated, pure Ni monolayer and pure Cu monolayer. The multilayer films consist of thin alternating Ni and Cu layers, and Ni is considerably harder than Cu (see Figure 4), but exhibits a lower friction force (Figure 6). Therefore, the lower dynamic friction force of the multilayer films can be attributed to neither the chemical composition nor the hardness (alone). According to tribological theory [24], the friction coefficient μ is proportional to the ratio of the shear strength τ to the hardness H. A low μ can be achieved with increasing H and decreasing τ (i.e., μ ∞ τ/H). Microscopically, shear of the Cu/Ni multilayer films should occur in the relatively weak Cu layer and, hence, τ is expected to be the value associated with Cu. Figure 4 showed that, for a fixed modulation period, the microhardness of the multilayer films (developed in this study) increased with increasing layer-thickness ratio of Ni to Cu. Accordingly, the ratio of τ to H (and, hence, the friction) would decrease with increasing Ni ratio of the Cu/Ni multilayer films. 

### 4.4. FW of Cu/Ni Multilayer Films 

As Figure 8a shows, the FW of Ti-811 against a Ti-811 ball is characterised by severe adhesion and cracking. This results mainly from, as discussed above, the 100% metallurgical compatibility between the Ti pad and Ti specimen, which leads to severe adhesion and a correspondingly high friction force between the self-matching articulating surfaces. The large tangential force is expected to contribute to the cracking observed in the FW scar (see Figure 8a). 

When the specimen surfaces were coated with a Cu/Ni multilayer film, the friction force decreased significantly (see Section 4.2). The amplitude of the alternating tangential stress in the contact region also decreased and the stress condition at the fretting contact surface was less severe than that associated with the non-coated surfaces. Accordingly, the fretting damage was reduced, as evidenced by the FW morphologies shown in Figure 8. Moreover, the increase in hardness of the Cu/Ni multilayer films with increasing Ni ratio of the film (Figure 4) may have contributed to the enhanced FW resistance. This stems from the fact that increased hardness may result in reduced abrasive damage of FW debris trapped in the articulating surfaces during FW. 

### 4.5. FF of Cu/Ni Multilayer Films 

As shown in Table 2, the FFR of the Ti-811 alloy can be improved by coating the alloy with a pure Ni or Cu layer, but the latter yields far greater improvement than the former. The metallurgical compatibility between Ti and Ni is larger than that between Ti and Cu. Therefore, as previously discussed, stronger adhesion and, hence, a higher friction force (Figure 6) are expected for the Ti pad-Ni film pair compared with that occurring for the Ti pad-Cu film pair; hence, owing to its weak adhesion and low friction, Cu is more effective in increasing the FFL of the Ti-811 alloy.

Table 2 reveals that the FFR of the Ti-811 alloy has been effectively improved by the Cu/Ni multilayer films studied. This improvement is explained as follows (i) the amplitude of the alternating tangential stress in the contact region was reduced, owing to the good solid-lubrication capacity of these films and the consequent reduction in the friction force and (ii) due to the lattice misfit produced during the epitaxial growth process of the multilayer films, an alternating stress field was generated in the interfaces between the Cu and Ni layers, which acted as pinning sites for dislocation movement. Crack initiation and the driving force for crack propagation were both reduced, owing to these interfaces. The consequent passivation of the crack tip and deviation in the direction of crack propagation resulted in a high fracture toughness [25] and improved FFR. 

The FFR of the Cu/Ni multilayer films exhibits a strong dependence on the modulation ratio (see Table 2). According to FF theory, the FFR of a surface is strongly correlated with the FW (i.e., resistance to crack formation) and toughness (i.e., resistance to fatigue crack propagation) of the surface. 

Cu/Ni multilayer films with a modulation ratio of 3:1 (Cu_150nm_Ni_50nm_) had low hardness and bearing capacity (Figure 8b) and, hence, these films were easily damaged during the fretting contact. In this case, the multilayer could no longer provide effective protection against fretting and the severe FW (Figure 9c) promoted rapid propagation of fatigue cracks during the FF process, thereby leading to short FFL. 

For a given modulation, increasing the Ni ratio leads to enhanced FW resistance of the multilayer films with high hardness (this is especially true for the Cu_50nm_Ni_150nm_ film). However, the FFL of the Cu/Ni multilayer Cu_50nm_Ni_150nm_-coated Ti-811 alloy is shorter than that of the Cu_100nm_Ni_100nm_-coated alloy (i.e., alloy coated by multilayer with a modulation ratio of 1:1). This results mainly from the increasing FW resistance (Figure 8d) that, for a fixed modulation, occurs at high Ni ratios (Cu_50nm_Ni_150nm_) of the multilayer film. The toughness decreased significantly with an increasing Ni ratio, as evidenced by the occurrence of severe radial cracks (see Figure 5c) and cracks in the FF scar (Figure 9d). Decreasing fracture toughness of the multilayer films with increasing hardness is expected, as the toughness varies inversely with the square root of the hardness [26]. This decrease results in relatively easy crack initiation and propagation. Therefore, the highest FFR is obtained for the film with a modulation ratio of 1:1 (Cu_100nm_Ni_100nm_), which has a high FW resistance (Figure 8c) and the highest toughness among the three Cu/Ni multilayer films (Figure 5b).

## 5. Conclusions

Ion-assisted magnetron sputtering was used to deposit Cu/Ni multilayer films, with different modulation ratios, on a Ti-811 alloy and the effect of these films on the fretting damage behaviour of the alloy was investigated. The deposited multilayer films were dense, crack-free, and strongly bonded to the substrate. For a fixed modulation period, the microhardness of the films increased with increasing layer-thickness ratio of Ni to Cu, and films with a modulation ratio of 1:3 (Cu_50nm_Ni_150nm_) exhibited superhardness. 

Owing to the possibly of their excellent lubrication and friction-reduction properties (which resulted in reduced FW), and numerous interfaces that retard/stop crack initiation and propagation, the multilayer films all yielded improved FW resistance of the Ti-811 alloy. In general, the FW resistance of the Cu/Ni multilayer-film-coated Ti-811 alloy increased monotonically with the Ni ratio of the films.

However, the FFR of the coated Ti-811 alloy increased non-monotonically with the Ni ratio of the films. The film with a modulation ratio of 1:1 (Cu_100nmNi100nm_) had the highest FFR among the multilayer films, owing mainly to its high toughness and good FW resistance. This indicates that the factors affecting, and the mechanisms governing, FW differ from those associated with FF.

## Figures and Tables

**Table 1:** Residual stresses determined using an X-ray diffraction residual stress analyser.

Sample	Cu/MPa	Ni/MPa
Cu	−152 ± 39	–
Ni	–	−146 ± 28
Cu_150nm_Ni_50nm_	−782 ± 69	−664 ± 43
Cu_100nm_Ni_100nm_	−861 ± 42	−622 ± 58
Cu_50nm_Ni_150nm_	−929 ± 56	−575 ± 32

**Table 2:** Fretting fatigue lives of the Ti-811 alloy coated with different films.

Sample	Each Specimen FF Life	Average FF Life
Ti-811 alloy	95,102	88,207
	97,317	
	72,201	
Cu	268,737	259,148
	236,346	
	272,360	
Ni	89,213	107,628
	120,445	
	113,227	
Cu_150nm_Ni_50nm_	191,446	182,639
	195,213	
	161,260	
Cu_100nm_Ni_100nm_	446,439	435,842
	444,616	
	416,470	
Cu_50nm_Ni_150nm_	305,248	294,635
	300,216	
	278,442	

**Table 3:** EDX-determined elemental composition of the fretted zone (at%).

Sample	O	Al	Ti	Ni	Cu	V
Ti-811 alloy	54.09	–	44.63	–	–	1.28
Cu_150nm_Ni_50nm_	28.26	1.63	8.99	22.87	38.25	–
Cu_100nm_Ni_100nm_	24.27	1.14	6.17	32.45	35.97	–
Cu_50nm_Ni_150nm_	19.98	2.79	9.91	42.36	24.96	–

## References

[1] James C., Edgar A. (2003). Progress in structural materials for aerospace systems. Acta Mater..

[2] Zhou Y., Zeng W., Yu H. (2005). An investigation of a new near-beta forging process for titanium alloys and its application in aviation components. Mater. Sci. Eng. A.

[3] Wei D., Wang Y., Yang X. (2011). Analysis of failure behaviors of dovetail assemblies due to high gradient stress under contact loading. Eng. Fail. Anal..

[4] Lee B., Suh J., Lee H. (2011). Investigations on fretting fatigue in aircraft engine compressor blade. Eng. Fail. Anal..

[5] Waterhouse R.B. (1972). Fretting Corrosion.

[6] Liu K., Hill M. (2009). The effects of laser peening and shot peening on fretting fatigue in Ti-6Al-4V coupons. Tribol. Int..

[7] Golden P., Hutson A., Sundaram V. (2007). Effect of surface treatments on fretting fatigue of Ti-6Al-4V. Int. J. Fatigue.

[8] Tang J., Liu D., Zhang X. (2016). Effects of Plasma ZrN Metallurgy and Shot Peening Duplex Treatment on Fretting Wear and Fretting Fatigue Behavior of Ti6Al4V Alloy. Materials.

[9] Du D., Liu D., Ye Z. (2014). Fretting wear and fretting fatigue behaviors of diamond-like carbon and graphite-like carbon films deposited on Ti-6Al-4V alloy. Appl. Surf. Sci..

[10] Yu S., Liu D., Zhang X. (2015). Effects of combined plasma chromizing and shot peening on the fatigue properties of a Ti6Al4V alloy. Appl. Surf. Sci..

[11] Tang C., Liu D., Tang B. (2016). Influence of plasma molybdenizing and shot-peening on fretting damage behavior oftitanium alloy. Appl. Surf. Sci..

[12] Zhou Y., Rao G., Wang J. (2011). Influence of Ti/TiN bilayered and multilayered films on the axial fatigue performance of Ti46Al8Nb alloy. Thin Solid Films.

[13] Zhang Z., Rapaud O., Allain N. (2009). Microstructures and tribological properties of CrN/ZrN nanoscale multilayer coatings. Appl. Surf. Sci..

[14] LiU Y., Bufford D., Wang H. (2011). Mechanical properties of highly textured Cu/Ni multilayers. Acta Mater..

[15] Abadias G., Michel G., Tromas C. (2007). Stress, interfacial effects and mechanical properties of nanoscale multilayered coatings. Surf. Coat. Technol..

[16] Zhang G., Wu Z., Wang M. (2007). Structure evolution and mechanical properties enhancement of Al/AlN multilayer. Appl. Surf. Sci..

[17] Zhang G., Wang T., Chen H. (2015). Microstructure, mechanical and tribological properties of TiN/Mo_2_N nano-multilayerfilms deposited by magnetron sputtering. Surf. Coat. Technol..

[18] Zhang X., Liu D. (2011). Improvement of the fretting damage resistance of Ti-811 alloy by Cu/Ni multilayer films. Tribol. Int..

[19] Ghosh S., Limaye P., Bhattachary S. (2007). Effect of Ni sublayer thickness on sliding wear characteristics of electrodeposited Ni/Cu multilayer coatings. Surf. Coat. Technol..

[20] Burnett P., Rickerby D. (1987). The mechanical properties of wear-resistant coatings I: Modelling of hardness behaviour. Thin Solid Films.

[21] Carpenter J.S., Misra A., Anderson P.M. (2012). Achieving maximum hardness in semi-coherent multilayer thin films with unequal layer thickness. Acta Mater..

[22] Abadias G., Uglov V., Saladukhin I. (2016). Growth, structural and mechanical properties of magnetron-sputtered ZrN_SiNx nanolaminated coatings. Surf. Coat. Technol..

[23] Rabinowicz E. (1995). Friction and Wear of Materials.

[24] Bowden F., Moore A., Tabor D. (1943). The Ploughing and Adhesion of Sliding Metals. J. Appl. Phys..

[25] Stoudt M.R., Ricker R.E., Cammarat R.C. (2001). The influence of a multilayered metallic coating on fatigue crack nucleation. Int. J. Fatigue.

[26] Wolfe D., Singh J., Narasimhan K. (2002). Synthesis of titanium carbide/chromium carbide multilayers by the co-evaporation of multiple ingots by electron beam physical vapor deposition. Surf. Coat. Technol..

